# 2-Isopropoxyphenyl *N*-methyl­carbamate

**DOI:** 10.1107/S1600536809002372

**Published:** 2009-01-23

**Authors:** Jun Wu, Min-Hao Xie, Shi-Neng Luo, Pei Zou, Yong-Jun He

**Affiliations:** a Jiangsu Institute of Nuclear Medicine, Wuxi 214063, People’s Republic of China

## Abstract

In the title compound, C_11_H_15_NO_3_, the mean planes of the carboxamide and isopropyl groups are inclined at 109.9 (1) and 128.7 (2)°, respectively, to the mean plane of the phen­oxy group. In the crystal structure, mol­ecules are stacked along the *b* axis, without any π–π inter­actions. The stacked columns are linked together by inter­molecular N—H⋯O hydrogen bonds, with an N⋯O distance of 2.842 (2) Å.

## Related literature

For background literature, see: Abburi & Nutalapati (2004 ▶); Moreno *et al.* (2001 ▶); Wang *et al.* (1998 ▶). For a report of a similar compound, see: Czugler & Kalman (1975 ▶).
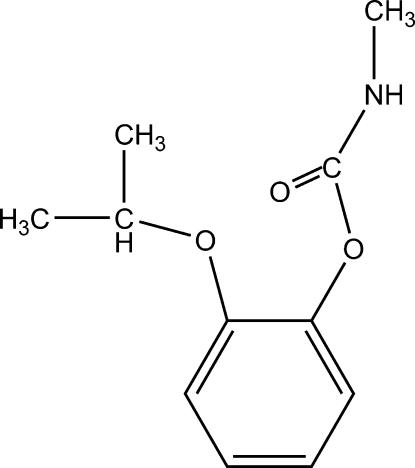


## Experimental

### 

#### Crystal data

C_11_H_15_NO_3_
*M* *_r_* = 209.24
Monoclinic, 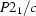

*a* = 13.275 (3) Å
*b* = 8.8890 (18) Å
*c* = 9.931 (2) Å
β = 90.59 (3)°
*V* = 1171.8 (4) Å^3^
*Z* = 4
Mo *K*α radiation
μ = 0.09 mm^−1^
*T* = 293 (2) K
0.30 × 0.10 × 0.10 mm

#### Data collection

Enraf–Nonius CAD-4 diffractometer
Absorption correction: ψ scan (*CAD-4 Software*; Enraf–Nonius, 1989 ▶) *T*_min_ = 0.975, *T*_max_ = 0.991
2257 measured reflections
2121 independent reflections
1255 reflections with *I* > 2σ(*I*)
*R*_int_ = 0.023
3 standard reflections every 200 reflections intensity decay: 1%

#### Refinement

*R*[*F* ^2^ > 2σ(*F* ^2^)] = 0.060
*wR*(*F* ^2^) = 0.152
*S* = 1.01
2121 reflections
136 parameters
H-atom parameters constrained
Δρ_max_ = 0.21 e Å^−3^
Δρ_min_ = −0.16 e Å^−3^

### 

Data collection: *CAD-4 Software* (Enraf–Nonius, 1989 ▶); cell refinement: *CAD-4 Software*; data reduction: *XCAD4* (Harms & Wocadlo, 1995 ▶); program(s) used to solve structure: *SHELXS97* (Sheldrick, 2008 ▶); program(s) used to refine structure: *SHELXL97* (Sheldrick, 2008 ▶); molecular graphics: *SHELXTL* (Sheldrick, 2008 ▶); software used to prepare material for publication: *SHELXTL*.

## Supplementary Material

Crystal structure: contains datablocks I, global. DOI: 10.1107/S1600536809002372/pv2125sup1.cif

Structure factors: contains datablocks I. DOI: 10.1107/S1600536809002372/pv2125Isup2.hkl

Additional supplementary materials:  crystallographic information; 3D view; checkCIF report

## Figures and Tables

**Table 1:** Hydrogen-bond geometry (Å, °)

*D*—H⋯*A*	*D*—H	H⋯*A*	*D*⋯*A*	*D*—H⋯*A*
N1—H0*A*⋯O3^i^	0.86	2.02	2.842 (2)	159

Symmetry code: (i) 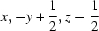.

**Fig. 1.**
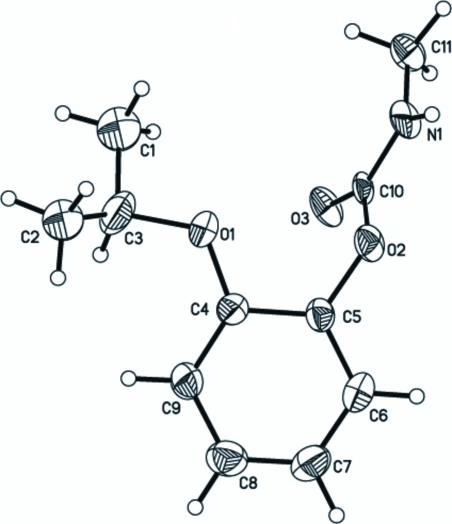
A view of the title compound with the atomic numbering scheme. Displacement ellipsoids were drawn at the 30% probability level.

## supplementary crystallographic information

### Comment

The title compound is one of the most important carbamate pesticides. It is widely used to control agricultural and household insect pests due to its low toxicity to mammals and other vertebrates (Abburi & Nutalapati, 2004; Moreno *et al.*, 2001; Wang *et al.*, 1998). We report here the crystal structure of title compound, (I).

The bond lengths and bond angles in title molecule (Fig. 1) are in agreement with those reported for a similar compound incorporating the phenoxycarboxamide group (Czugler & Kalman, 1975). In (I), the O3/C10/N1/C11 plane forms a dihedral angle of 109.9 (1)° with the C4–C9/O2 plane. The C1/C2/C3 plane forms a dihedral angle of 128.7 (2)° with the C4–C9/O1 plane. In the crystal structure, the molecules are stacked along the *b* axis, without any π–π interaction. The stacked columns are linked together by the intermolecular hydrogen bonds of the type N—- H···O, details have been given in Table 1.

### Experimental

A sample of commercial 2-(1-methylethoxy)phenol methylcarbamate (Aldrich) was crystallized by slow evaporation of a solution in acetone.

### Refinement

Positional parameters of all the H atoms bonded to C atoms were calculated geometrically and were allowed to ride on the C atoms to which they are bonded, with N—H = 0.86 and C—H = 0.93, 0.96 and 0.98 Å for aryl, methyl and methine H atoms and *U*_iso_(H) = 1.5*U*_eq_(methyl) and 1.2*U*_eq_(the rest) parent atoms.

### Crystal data

**Table:**

C_11_H_15_NO_3_	*F*(000) = 448
*M**_r_* = 209.24	*D*_x_ = 1.186 Mg m^−^^3^
Monoclinic, *P*2_1_/*c*	Mo *K*α radiation, λ = 0.71073 Å
Hall symbol: -P 2ybc	Cell parameters from 25 reflections
*a* = 13.275 (3) Å	θ = 9–12°
*b* = 8.8890 (18) Å	µ = 0.09 mm^−^^1^
*c* = 9.931 (2) Å	*T* = 293 K
β = 90.59 (3)°	Needle, colourless
*V* = 1171.8 (4) Å^3^	0.30 × 0.10 × 0.10 mm
*Z* = 4	

### Data collection

**Table:**

Enraf–Nonius CAD-4 diffractometer	1255 reflections with *I* > 2σ(*I*)
Radiation source: fine-focus sealed tube	*R*_int_ = 0.023
graphite	θ_max_ = 25.3°, θ_min_ = 1.5°
ω/2θ scans	*h* = −15→15
Absorption correction: ψ scan (*CAD-4 Software*; Enraf–Nonius, 1989)	*k* = 0→10
*T*_min_ = 0.975, *T*_max_ = 0.991	*l* = 0→11
2257 measured reflections	3 standard reflections every 200 reflections
2121 independent reflections	intensity decay: 1%

### Refinement

**Table:**

Refinement on *F*^2^	Secondary atom site location: difference Fourier map
Least-squares matrix: full	Hydrogen site location: difference Fourier map
*R*[*F*^2^ > 2σ(*F*^2^)] = 0.060	H-atom parameters constrained
*wR*(*F*^2^) = 0.152	*w* = 1/[σ^2^(*F*_o_^2^) + (0.06*P*)^2^ + 0.55*P*] where *P* = (*F*_o_^2^ + 2*F*_c_^2^)/3
*S* = 1.01	(Δ/σ)_max_ < 0.001
2121 reflections	Δρ_max_ = 0.21 e Å^−^^3^
136 parameters	Δρ_min_ = −0.16 e Å^−^^3^
0 restraints	Extinction correction: *SHELXL97* (Sheldrick, 2008)
Primary atom site location: structure-invariant direct methods	Extinction coefficient: none

### Special details

**Table:**

Geometry. All e.s.d.'s (except the e.s.d. in the dihedral angle between two l.s. planes) are estimated using the full covariance matrix. The cell e.s.d.'s are taken into account individually in the estimation of e.s.d.'s in distances, angles and torsion angles; correlations between e.s.d.'s in cell parameters are only used when they are defined by crystal symmetry. An approximate (isotropic) treatment of cell e.s.d.'s is used for estimating e.s.d.'s involving l.s. planes.
Refinement. Refinement of *F*^2^ against ALL reflections. The weighted *R*-factor *wR* and goodness of fit *S* are based on *F*^2^, conventional *R*-factors *R* are based on *F*, with *F* set to zero for negative *F*^2^. The threshold expression of *F*^2^ > σ(*F*^2^) is used only for calculating *R*-factors(gt) *etc*. and is not relevant to the choice of reflections for refinement. *R*-factors based on *F*^2^ are statistically about twice as large as those based on *F*, and *R*- factors based on ALL data will be even larger.

### Fractional atomic coordinates and isotropic or equivalent isotropic displacement parameters (Å^2^)

**Table:**

	*x*	*y*	*z*	*U*_iso_*/*U*_eq_	
N1	0.60393 (17)	0.3161 (3)	−0.19856 (18)	0.0506 (6)	
H0A	0.6177	0.2831	−0.2777	0.061*	
O1	0.82141 (16)	0.1745 (2)	0.0186 (2)	0.0748 (7)	
O2	0.66395 (14)	0.0939 (2)	−0.13306 (16)	0.0526 (5)	
O3	0.60236 (15)	0.2566 (2)	0.02273 (15)	0.0566 (6)	
C1	0.8569 (3)	0.4130 (4)	0.1055 (4)	0.0941 (12)	
H1A	0.7868	0.4283	0.1236	0.141*	
H1B	0.8712	0.4451	0.0154	0.141*	
H1C	0.8968	0.4705	0.1682	0.141*	
C2	0.9872 (3)	0.2104 (4)	0.1067 (4)	0.0874 (11)	
H2A	0.9973	0.1079	0.1341	0.131*	
H2B	1.0274	0.2756	0.1625	0.131*	
H2C	1.0067	0.2218	0.0144	0.131*	
C3	0.8819 (3)	0.2495 (4)	0.1202 (4)	0.0884 (12)	
H3A	0.8594	0.2159	0.2089	0.106*	
C4	0.7804 (2)	0.0381 (3)	0.0493 (3)	0.0497 (7)	
C5	0.6971 (2)	−0.0015 (3)	−0.0310 (2)	0.0448 (7)	
C6	0.6512 (2)	−0.1395 (4)	−0.0155 (3)	0.0587 (8)	
H6A	0.5964	−0.1656	−0.0696	0.070*	
C7	0.6867 (3)	−0.2379 (4)	0.0800 (3)	0.0692 (9)	
H7A	0.6564	−0.3316	0.0899	0.083*	
C8	0.7652 (3)	−0.1993 (4)	0.1594 (4)	0.0707 (9)	
H8A	0.7882	−0.2667	0.2244	0.085*	
C9	0.8130 (2)	−0.0602 (3)	0.1463 (3)	0.0668 (9)	
H9A	0.8664	−0.0347	0.2030	0.080*	
C10	0.62194 (18)	0.2312 (3)	−0.0929 (2)	0.0386 (6)	
C11	0.5614 (2)	0.4638 (3)	−0.1832 (3)	0.0588 (8)	
H11A	0.5510	0.5082	−0.2704	0.088*	
H11B	0.6067	0.5254	−0.1313	0.088*	
H11C	0.4981	0.4564	−0.1378	0.088*	

### Atomic displacement parameters (Å^2^)

**Table:**

	*U*^11^	*U*^22^	*U*^33^	*U*^12^	*U*^13^	*U*^23^
N1	0.0653 (15)	0.0723 (17)	0.0143 (9)	−0.0010 (13)	0.0002 (9)	−0.0007 (9)
O1	0.0689 (15)	0.0644 (14)	0.0905 (15)	−0.0241 (12)	−0.0349 (12)	0.0110 (12)
O2	0.0634 (13)	0.0637 (13)	0.0305 (9)	0.0012 (10)	−0.0072 (8)	−0.0068 (9)
O3	0.0708 (14)	0.0784 (14)	0.0206 (9)	0.0144 (11)	−0.0055 (8)	−0.0029 (9)
C1	0.103 (3)	0.081 (3)	0.097 (3)	−0.006 (2)	0.004 (2)	−0.004 (2)
C2	0.076 (3)	0.095 (3)	0.090 (3)	−0.004 (2)	0.001 (2)	−0.001 (2)
C3	0.080 (3)	0.066 (2)	0.119 (3)	−0.022 (2)	−0.028 (2)	−0.016 (2)
C4	0.0456 (16)	0.0444 (17)	0.0590 (17)	−0.0041 (14)	−0.0059 (13)	0.0040 (13)
C5	0.0429 (15)	0.0550 (18)	0.0365 (13)	−0.0029 (14)	−0.0024 (12)	−0.0027 (12)
C6	0.0532 (19)	0.064 (2)	0.0585 (18)	−0.0117 (16)	−0.0025 (14)	−0.0144 (16)
C7	0.070 (2)	0.0504 (19)	0.087 (2)	−0.0091 (17)	0.0016 (19)	−0.0051 (18)
C8	0.071 (2)	0.057 (2)	0.084 (2)	0.0083 (18)	−0.0022 (18)	0.0126 (17)
C9	0.056 (2)	0.055 (2)	0.088 (2)	0.0049 (16)	−0.0258 (17)	0.0023 (17)
C10	0.0387 (14)	0.0598 (17)	0.0171 (11)	−0.0121 (13)	−0.0076 (9)	0.0017 (11)
C11	0.066 (2)	0.069 (2)	0.0413 (15)	0.0057 (17)	−0.0028 (14)	0.0027 (14)

### Geometric parameters (Å, °)

**Table:**

N1—C10	1.313 (3)	C2—H2C	0.9600
N1—C11	1.438 (4)	C3—H3A	0.9800
N1—H0A	0.8600	C4—C9	1.368 (4)
O1—C4	1.365 (3)	C4—C5	1.402 (4)
O1—C3	1.446 (4)	C5—C6	1.379 (4)
O2—C5	1.390 (3)	C6—C7	1.370 (4)
O2—C10	1.401 (3)	C6—H6A	0.9300
O3—C10	1.201 (3)	C7—C8	1.346 (4)
C1—C3	1.498 (5)	C7—H7A	0.9300
C1—H1A	0.9600	C8—C9	1.395 (4)
C1—H1B	0.9600	C8—H8A	0.9300
C1—H1C	0.9600	C9—H9A	0.9300
C2—C3	1.448 (5)	C11—H11A	0.9600
C2—H2A	0.9600	C11—H11B	0.9600
C2—H2B	0.9600	C11—H11C	0.9600
			
C10—N1—C11	120.6 (2)	C9—C4—C5	118.8 (3)
C10—N1—H0A	119.7	C6—C5—O2	119.2 (2)
C11—N1—H0A	119.7	C6—C5—C4	120.5 (3)
C4—O1—C3	118.3 (2)	O2—C5—C4	120.2 (2)
C5—O2—C10	116.62 (17)	C7—C6—C5	119.8 (3)
C3—C1—H1A	109.5	C7—C6—H6A	120.1
C3—C1—H1B	109.5	C5—C6—H6A	120.1
H1A—C1—H1B	109.5	C8—C7—C6	120.1 (3)
C3—C1—H1C	109.5	C8—C7—H7A	120.0
H1A—C1—H1C	109.5	C6—C7—H7A	120.0
H1B—C1—H1C	109.5	C7—C8—C9	121.4 (3)
C3—C2—H2A	109.5	C7—C8—H8A	119.3
C3—C2—H2B	109.5	C9—C8—H8A	119.3
H2A—C2—H2B	109.5	C4—C9—C8	119.4 (3)
C3—C2—H2C	109.5	C4—C9—H9A	120.3
H2A—C2—H2C	109.5	C8—C9—H9A	120.3
H2B—C2—H2C	109.5	O3—C10—N1	128.1 (3)
O1—C3—C2	110.8 (3)	O3—C10—O2	121.8 (2)
O1—C3—C1	105.0 (3)	N1—C10—O2	110.05 (19)
C2—C3—C1	115.9 (3)	N1—C11—H11A	109.5
O1—C3—H3A	108.3	N1—C11—H11B	109.5
C2—C3—H3A	108.3	H11A—C11—H11B	109.5
C1—C3—H3A	108.3	N1—C11—H11C	109.5
O1—C4—C9	127.0 (3)	H11A—C11—H11C	109.5
O1—C4—C5	114.2 (2)	H11B—C11—H11C	109.5
			
C4—O1—C3—C2	91.9 (4)	C4—C5—C6—C7	0.6 (4)
C4—O1—C3—C1	−142.3 (3)	C5—C6—C7—C8	0.9 (5)
C3—O1—C4—C9	−22.3 (5)	C6—C7—C8—C9	−0.6 (5)
C3—O1—C4—C5	158.6 (3)	O1—C4—C9—C8	−176.6 (3)
C10—O2—C5—C6	116.3 (3)	C5—C4—C9—C8	2.5 (5)
C10—O2—C5—C4	−67.7 (3)	C7—C8—C9—C4	−1.1 (5)
O1—C4—C5—C6	176.9 (2)	C11—N1—C10—O3	4.4 (4)
C9—C4—C5—C6	−2.3 (4)	C11—N1—C10—O2	−179.5 (2)
O1—C4—C5—O2	1.0 (4)	C5—O2—C10—O3	−10.2 (3)
C9—C4—C5—O2	−178.1 (3)	C5—O2—C10—N1	173.3 (2)
O2—C5—C6—C7	176.5 (3)		

### Hydrogen-bond geometry (Å, °)

**Table:**

*D*—H···*A*	*D*—H	H···*A*	*D*···*A*	*D*—H···*A*
N1—H0A···O3^i^	0.86	2.02	2.842 (2)	159

Symmetry codes: (i) *x*, −*y*+1/2, *z*−1/2.
